# Phytic Acid Maintains Peripheral Neuron Integrity and Enhances Survivability against Platinum-Induced Degeneration via Reducing Reactive Oxygen Species and Enhancing Mitochondrial Membrane Potential

**DOI:** 10.1021/acschemneuro.3c00739

**Published:** 2024-03-06

**Authors:** Arjun Prasad Tiwari, Bayne Albin, Khayzaran Qubbaj, Prashant Adhikari, In Hong Yang

**Affiliations:** Center for Biomedical Engineering and Science, Department of Mechanical Engineering and Engineering Science, University of North Carolina at Charlotte, Charlotte, North Carolina 28223, United States

**Keywords:** cisplatin, peripheral neuropathy, phytic acid, neuronal survivability, reactive oxygen species, mitochondrial membrane potential

## Abstract

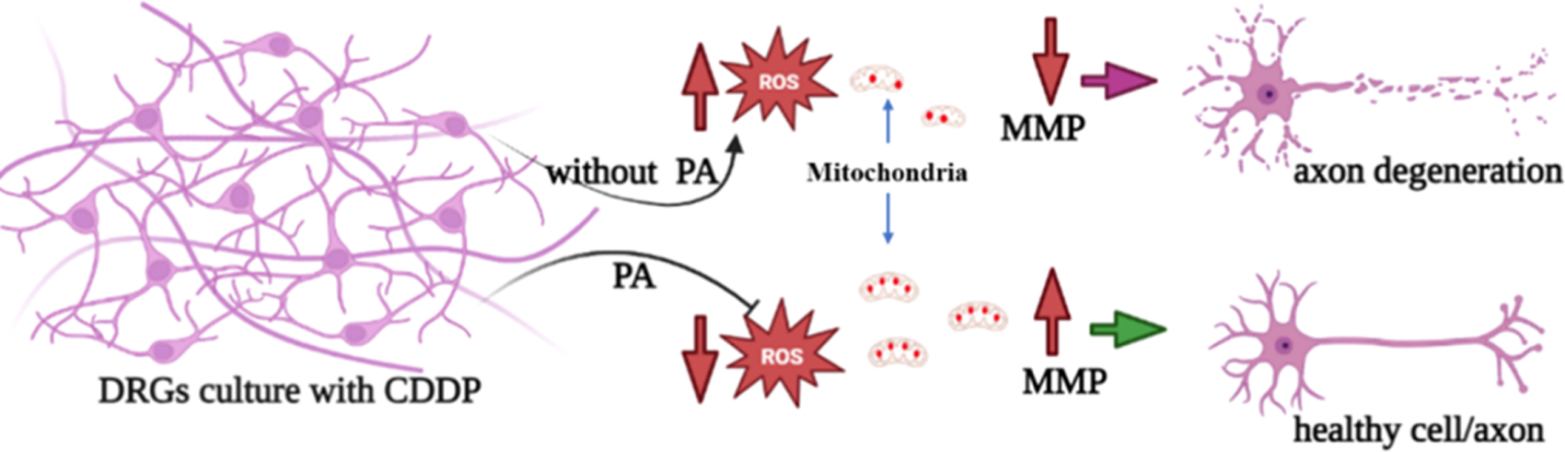

Phytic acid (PA) has been reported to possess anti-inflammatory and antioxidant properties that are critical for neuroprotection in neuronal disorders. This raises the question of whether PA can effectively protect sensory neurons against chemotherapy-induced peripheral neuropathy (CIPN). Peripheral neuropathy is a dose-limiting side effect of chemotherapy treatment often characterized by severe and abnormal pain in hands and feet resulting from peripheral nerve degeneration. Currently, there are no effective treatments available that can prevent or cure peripheral neuropathies other than symptomatic management. Herein, we aim to demonstrate the neuroprotective effects of PA against the neurodegeneration induced by the chemotherapeutics cisplatin (CDDP) and oxaliplatin. Further aims of this study are to provide the proposed mechanism of PA-mediated neuroprotection. The neuronal protection and survivability against CDDP were characterized by axon length measurements and cell body counting of the dorsal root ganglia (DRG) neurons. A cellular phenotype study was conducted microscopically. Intracellular reactive oxygen species (ROS) was estimated by fluorogenic probe dichlorofluorescein. Likewise, mitochondrial membrane potential (MMP) was assessed by fluorescent MitoTracker Orange CMTMRos. Similarly, the mitochondria-localized superoxide anion radical in response to CDDP with and without PA was evaluated. The culture of primary DRG neurons with CDDP reduced axon length and overall neuronal survival. However, cotreatment with PA demonstrated that axons were completely protected and showed increased stability up to the 45-day test duration, which is comparable to samples treated with PA alone and control. Notably, PA treatment scavenged the mitochondria-specific superoxide radicals and overall intracellular ROS that were largely induced by CDDP and simultaneously restored MMP. These results are credited to the underlying neuroprotection of PA in a platinum-treated condition. The results also exhibited that PA had a synergistic anticancer effect with CDDP in ovarian cancer in vitro models. For the first time, PA’s potency against CDDP-induced PN is demonstrated systematically. The overall findings of this study suggest the application of PA in CIPN prevention and therapeutic purposes.

## 1 Introduction

Cisplatin (CDDP) is a platinum-based compound and has been used for treating a wide range of cancers including ovarian,^1^ testicular,^2,3^ bladder,^4^ lung,^5^ and breast cancers.^6^ However, treatment with CDDP can cause many serious side effects, including kidney damage, hearing loss, and peripheral neuropathy (PN).^7,8^ On the other hand, oxaliplatin is another potent platinum-based chemotherapeutic agent that has been used to treat colorectal cancer.^9,10^ Nevertheless, oxaliplatin also induces PN in patients in a dose-dependent manner.^10^ PN is a serious adverse effect that occurs during platinum drug treatment or even after the treatment has been stopped.^3^ PN is manifested by numbness, tingling, burning sensation, and weakness in hands and feet.^3^ Over time, it can progress to a painful and debilitating condition that can greatly affect a patient’s quality of life. The onset of PN is common when patients receive cumulative doses exceeding 300 mg/m^2^ in the case of CDDP administration.^11,12^ On the other hand, oxaliplatin-induced neurotoxicity consists of rapid onset sensory neuropathy and/or late onset neuropathy following multiple cycles of therapy.^10,13^ Unfortunately, there are currently no established drugs for the prevention and treatment of CIPN, according to the American Society of Clinical Oncology (ASCO) and the European Society of Medical Oncology (ESMO).^14,15^

CIPNs are often managed with dose reduction, dose delay, substitutions, and stopping chemotherapy.^14,15^ Duloxetine, an antidepressant drug, is a recommended drug for the treatment of established painful neuropathy.^14−16^ Despite the fact that it is not recommended for routine usage.^17^ CIPN management with this drug is solely symptomatic and has not been reported to have potential for peripheral nerve protection and regeneration, as far as we know. On the other hand, there has been increasing interest in finding new drug agents for CIPN treatment such as khellin,^18^ magnolol,^19^ quercetin,^20^ pterostilbene,^21^ dietary supplements,^22^ and pharmaceutical compounds.^23,24^ Nevertheless, clinical outcomes are not satisfactory in the studied groups receiving neurotoxic chemotherapy.^22^ The outlined pharmaceutical interventions or supplementary products generally do not show anticancer activities. Efforts to find new therapeutic drugs or repurpose the compounds for the elimination of potential side effects in cancer patients and survivors are critical for treatment development.

PA, a known and long-studied naturally occurring biocompound, also known as inositol hexaphosphate (IP6), is found in many plant-based foods, including grains, legumes, and nuts.^25,26^ There is evidence to suggest that PA may have anticancer properties.^27^ Similarly, PA is reported to have protective effects in neurological disorders such as Parkinson’s disease and cerebral injuries by attenuating inflammatory responses and decreasing oxidative damage.^28,29^ Significant protection of neuron cells by PA against a 6-hydroxydopamine-induced Parkinson cellular model via reducing reactive oxygen species (ROS) and oxidative mitochondrial damages has been reported.^30^

The onset of PN is a result of the accumulation of anticancer drugs in the nerves and interference with normal functionality by oxidative damage, inflammation, and mitochondrial dysfunction.^31^ Evidence from in vitro and in vivo data show that activation of proinflammatory genes TNF-α and NF-κB and excessive production of free radicals are critically involved in neuropathic pain.^32,33^ Mitochondrial damage is considered a key aspect due to the excessive intracellular ROS production that causes peripheral nerve degeneration.^34^ PA has the ability to accept or donate electrons.^35^ Herein, the ROS can be scavenged by PA, which reduces oxidative and mitochondrial damage caused by accumulated platinum drugs in axons. In this study, we envision protecting the sensory neurons that are highly vulnerable to degeneration by platinum drugs. There has not been any work that shows PA-mediated neuroprotection against CDDP treatment to date, as far as we know.

## 2 Results

### 2.1 PA Protects Axons from CDDP-Induced Degeneration

**Figure 1:**
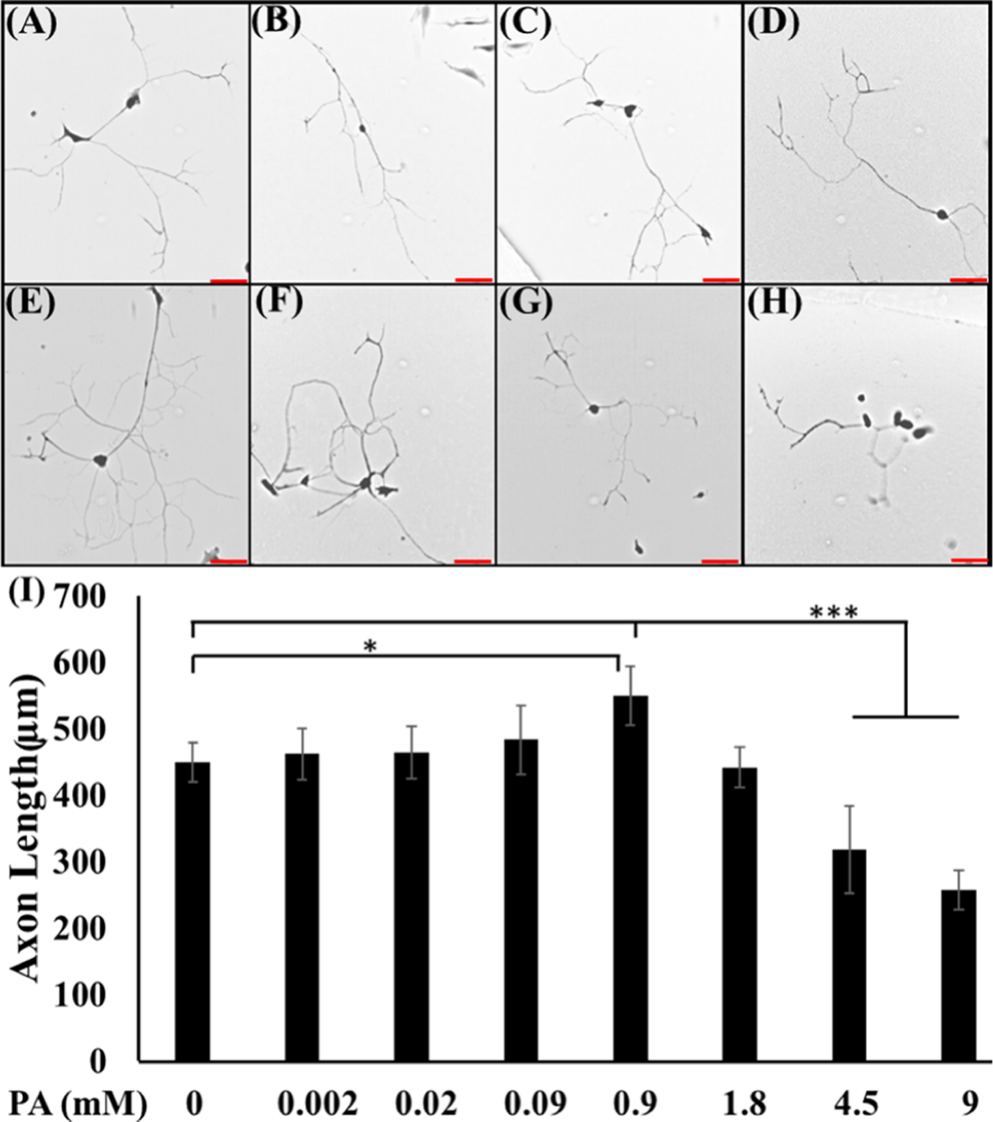
Effect of PA on axon length. Calcein staining fluorescence image of the DRG neurons treated with PA0 (control) (A), PA 0.002 (B), PA 0.02 (C), PA 0.09 (D), PA 0.9 (E), PA 1.8 (F), PA 4.5 (G), and PA 9 (H), and corresponding axon length data (I). The results are expressed as mean ± SE, *n* = 3. *P < 0.05, **P < 0.01, and ***P < 0.001. The scale bar is 100 μm. After 24 h of cell seeding, the cells were treated with CDDP, PA, and combination for 24 h before microscopy.

To investigate whether the PA coadministration protects CDDP-induced axon degeneration, dorsal root ganglia (DRG) cells were administered with PA along with CDDP and assessed based on axonal length measurement. As an optimization study, we have first evaluated the effect of varying PA concentrations on the DRG neurons. The fluorescence images showed axons and neurites without apparent blebbing of the cells treated up to 1.8 mM PA. However, the axons were shorter and fragmented with the cells treated with 4.5 and 9 mM PA (Figure 1A–H) compared to the control. Figure 1 I shows the axon length data quantitatively.

**Figure 2:**
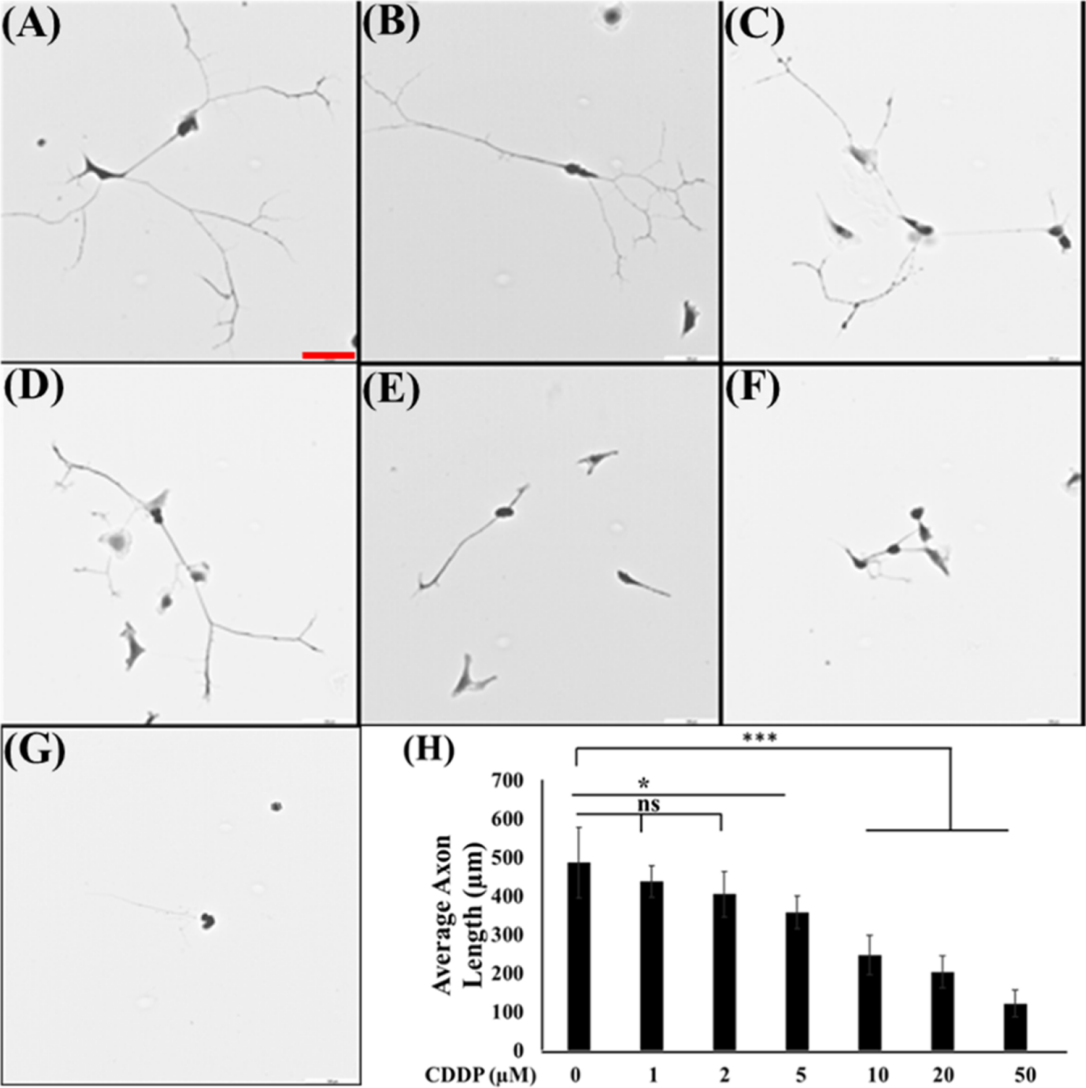
CDDP treatment on DRG neurons. Calcein staining fluorescence images of the DRG neurons treated with CDDP0 (control) (A), CDDP1μM (B), CDDP2μM (C), CDDP5μM (D), CDDP10μM (E), CDDP20μM (F), and CDDP50μM (G), and corresponding axon length data (H). The results are expressed as mean ± SE, *n* = 3. *P < 0.05, **P < 0.01, and ***P < 0.001, relative to control. The scale bar is 100 μm. Cells were treated with CDDP, PA, and combination for 24 h before microscopy.

**Figure 3:**
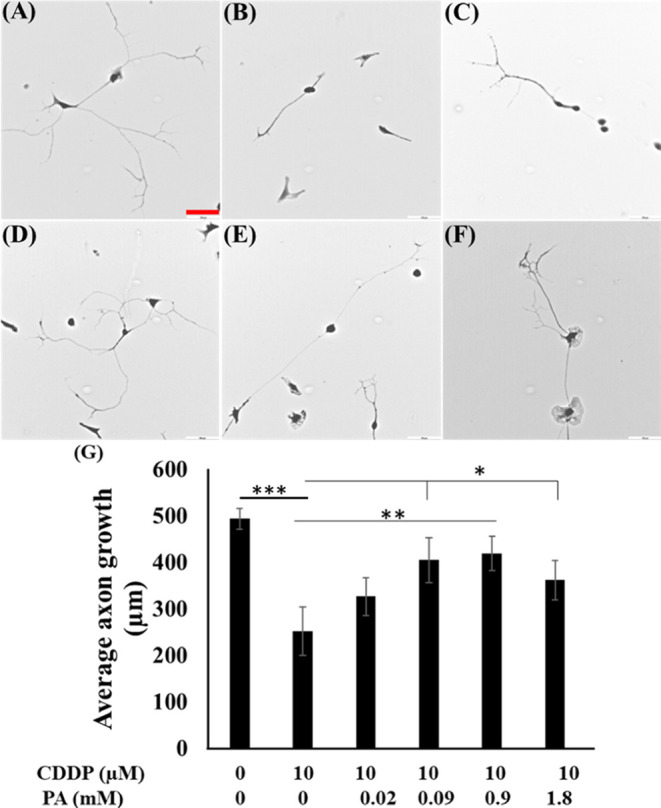
PA cotreatment on DRG neurons. Calcein staining fluorescence image of the DRG treated with control (A), CDDP10 (B), CDDP10PA0.02 (C), CDDP10PA0.09 (D), CDDP10PA0.9 (E), and CDDP10PA1.8 (F), and corresponding axon length data (G). The results are expressed as mean ± SE, *n* = 3. *P < 0.05, **P < 0.01, and ***P < 0.001, relative to control. The scale bar is 100 μm. Cells were treated with CDDP, PA, and combination for 24 h before microscopy.

The axon lengths with PA doses 0.002, 0.02, 0.09, 0.9, and 1.8 mM are comparable to the control (*P* > 0.05) while showing a decrease in the axon length with further increased PA concentration to 4.5 and 9 mM (*P* < 0.05). The sample treated with 0.9 mM had significantly longer axons compared to the control and others (*P* < 0.05). The DRG neurons treated with CDDP exhibited a gradual reduction in axon length in comparison to the control sample in a dose-dependent manner (Figure 2). The doses up to 2 μM did not have significant changes in the axon lengths compared to the control, but increased doses of 5 μM or more showed a sharp reduction in axon growth (control vs 5 μM-treated, *P* < 0.05; control vs 10, 20, and 50 μM-treated, *P* < 0.001). Interestingly, the introduction of PA alongside CDDP, regardless of concentration, inhibited CDDP-induced axon degeneration and exhibited a gradual increase of axon lengths with increasing PA concentration up to 0.9 mM (Figure 3). The microscopy image clearly shows the healthier cellular morphology with integrated axons with the PA cotreatment (Figure 3A–F). The results suggest that PA cotreatment with CDDP can prevent axonal degeneration caused by CDDP.

### 2.2 PA Cotreatment Improves the Survivability and Stability of Neurons

**Figure 4:**
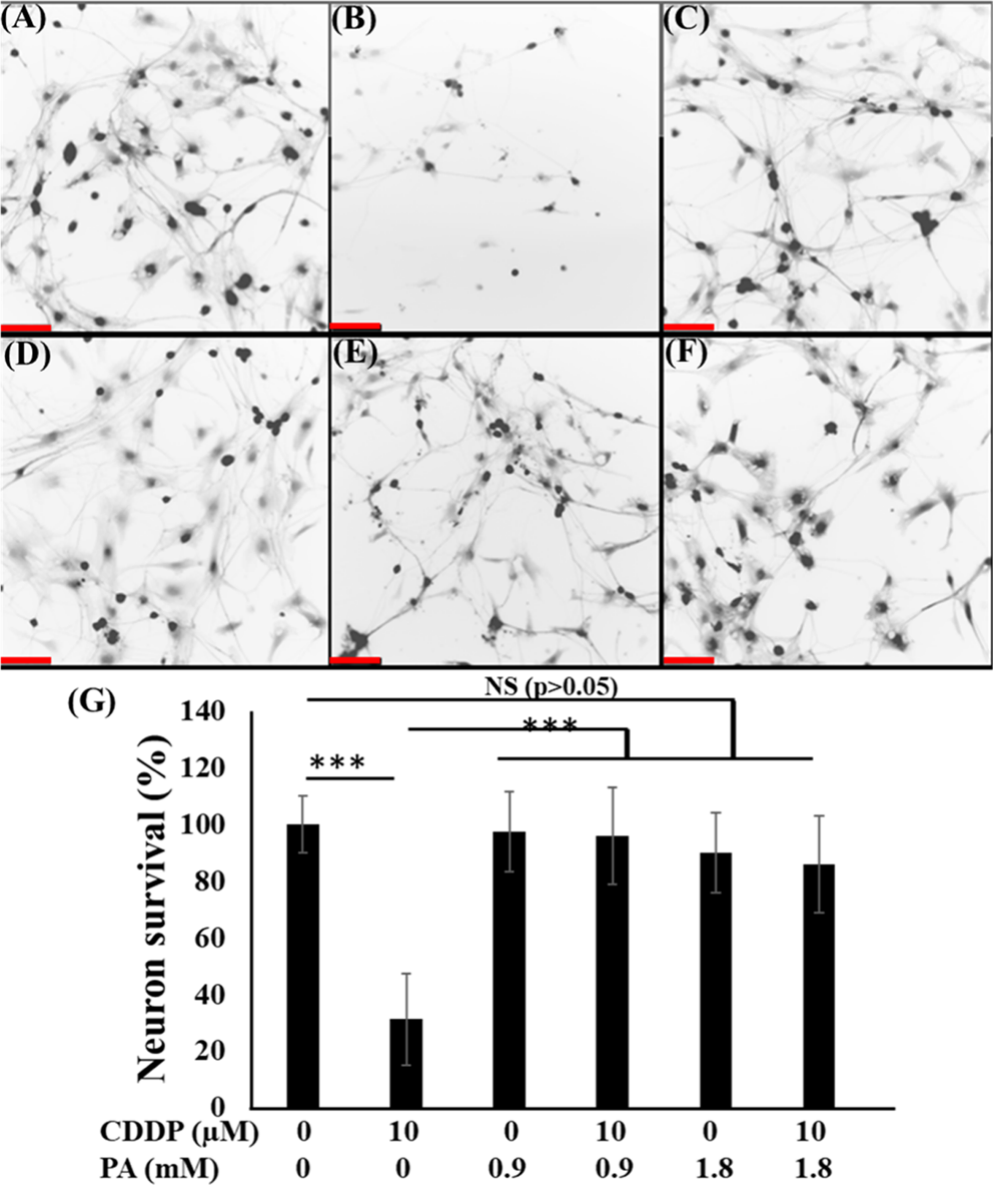
PA cotreatment on DRG neuron survivability. Calcein staining fluorescence images of the DRG neurons treated with control (A), CDDP10 (B), PA0.9 (C), CDDP10PA0.9 (D), PA1.8 (E), and CDDP10PA1.8 (F), and corresponding cell survival data (G). The results are expressed as mean ± SE, *n* = 3. *P < 0.05, **P < 0.01, and ***P < 0.001, relative to CDDP. The scale bar is 100 μm. Five-day-old DRGs were treated with drugs for another 7 days.

Neuronal survivability data showed that CDDP10 exposure led to only 26.2% cell survivability, which is significantly lower compared to PA alone or in combination. The PA 0.9 and 1.8 cotreatment had the cell survivability increased to 95.1 and 86.8%, respectively, achieving near-complete recovery, similar to control (*P* > 0.05; Figure 4G). On the other hand, PA had similar cell survivability as control samples (*P* > 0.05). The corresponding images reconfirmed that all soma and axons with PA or PA cotreatment were intact in contrast to CDDP-treated, where the axons were degenerated and lesser cells were found (Figure 4A–F).

**Figure 5:**
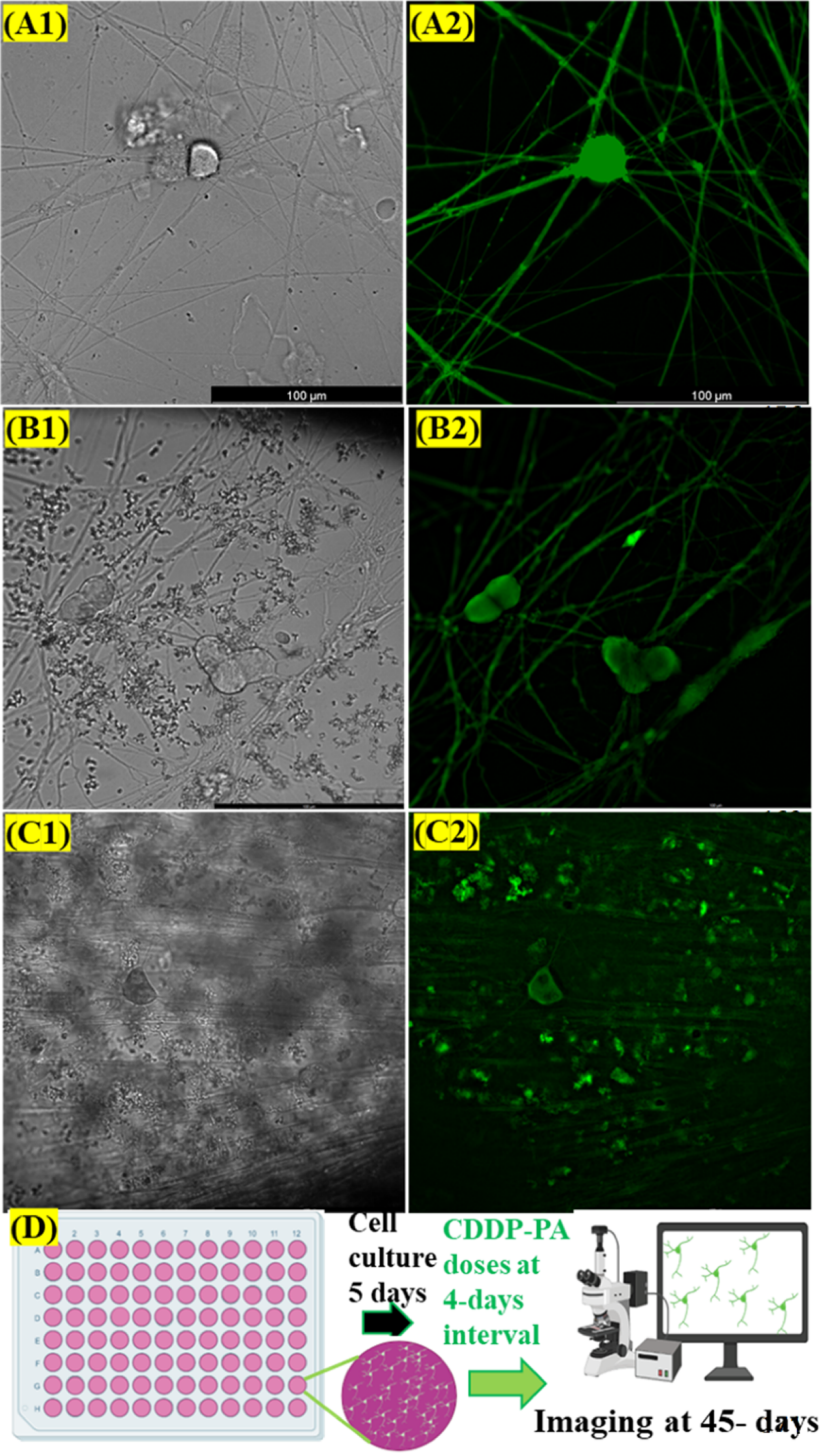
DRG cell stability study. Phase contrast and corresponding fluorescence images of the DRGs were taken 45 days following cotreatment. The images of DRG cells treated with CDDP0PA0 (control) (A1, A2), PA0.9 (B1, B2), and CDDP10PA0.9 (C1–C2), and illustration showing steps of the stability test (D). Numbers 1 and 2 in the images represent phase contrast and fluorescence images of corresponding samples, respectively. The scale bar is 100 μm. The drug treatment started on day 5 when the DRG axons confluently bonded over the substrate. The cultured medium containing drugs was replenished 50% by volume in 3-day intervals. The cells/axons treated with 10 μM CDDP were degraded at 14 days exposed time and therefore skipped treatment thereafter. The other samples were treated with PA and/or CDDP–PA combination. The numbers after CDDP represent μM, and the numbers after PA represent mM.

The response of PA cotreatment on neuronal stability was studied for up to 45 days through the qualitative changes of the cell’s phenotypes by microscopy. It is an established phenomenon that CDDP causes cell death and axon degeneration.^36^ Our results revealed that the cells treated with 10 μM CDDP led to reduced survivability to 26.2% at 7 days (Figure 4B,G). CDDP-exposed cells were already degenerated and killed completely within a 2-week period (Figure S1). Interestingly, the cells with PA cotreatment have shown resilience against CDDP-induced axonal degeneration for up to 45 days, comparable to the corresponding PA-treated and control samples (Figure 5). The cell bodies appeared oval and triangular shaped without noticeable damage, establishing healthy DRG cells phenotype.

### 2.3 PA’s Effect on CDDP’s Ability to Inhibit Cancer Cell Growth

**Figure 6:**
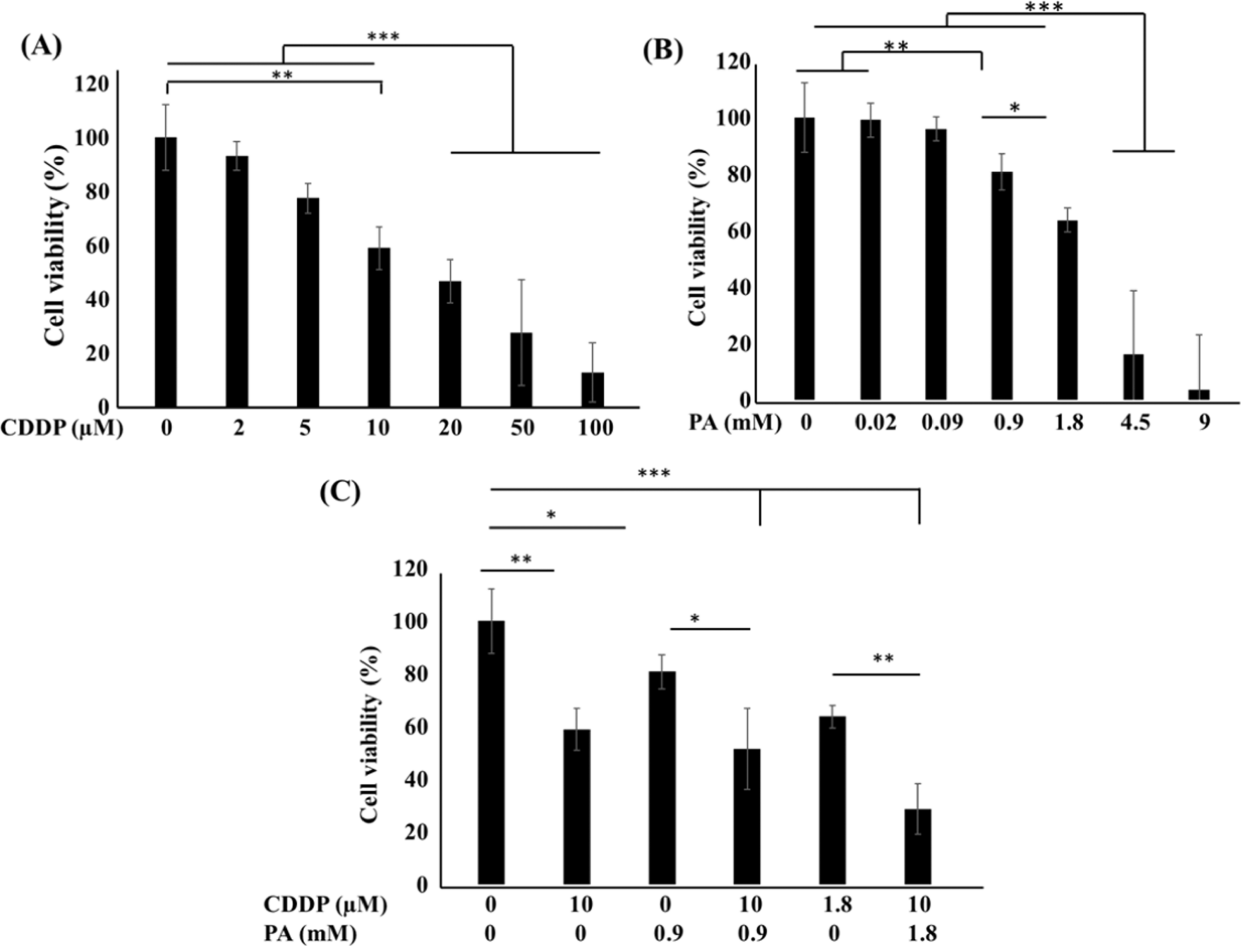
Effect of PA on cancer cell viability. The viability of SKOV-3 cells with the exposure of CDDP (A), PA (B), and CDDP–PA (C). Cell viability was measured after 48 h of treatment. The results are expressed as mean ± SE, *n* = 3. *P < 0.05, **P < 0.01, and ***P < 0.001.

Considering that PA could alter the anticancer activities of CDDP, we studied its effect on human ovarian cancer cells (SKOV-3). The results demonstrated that the CDDP reduced the SKOV-3 cell viability in a dose-dependent manner (Figure 6A). For instance, there was no obvious toxicity observed up to 2 μM; however, increased concentrations at 5 and 10 μM showed significantly reduced cell viability to 77.4 and 58.9%, which further reduced to 46.5, 27.3, and 12.8% for 20, 50, and 100 μM CDDP, respectively. The PA concentrations of 0.02 and 0.09 mM did not show toxicity on SKOV-3 cells. However, increasing cell death was recorded with gradually increasing concentration (Figure 6B). The combined effect of CDDP and PA is shown in Figure 6C. The cell viability was found to decrease with increasing PA concentrations. For instance, the 58.9% viability associated with CDDP10 alone, 80.1% viability of cells for PA0.9, and 63.7% cell viability for PA1.8 were noted to be reduced to 51.3 and 28.6%, respectively, when subjected to the combinatory approach. Results revealed that the PA concentration of equal or more than 0.9 mM had a synergistic effect on cancer cell death. The PA-cotreated cells are shown in Figure S2.

### 2.4 PA Cotreatment Scavenges the ROS Production and Maintains the MMP

Platinum drugs are known to enhance ROS production, which results in accelerating axon degeneration and other pathologies.^37^ In order to test whether PA has the inhibitory role of ROS and simultaneous neuroprotection, we assessed the intracellular ROS levels with fluorescein diacetate. The CDDP doses below 20 μM did not show any significant ROS signal when they were tested at 24 h. Herein, 50 μM CDDP was used for the ROS assessment. The results showed that 50 μM CDDP had a 2.7-fold increased ROS fluorescence signal compared to the control sample. However, ROS levels were retained to control levels when 0.09 and 0.9 mM PA were cotreated with CDDP50. To further confirm ROS inhibition by PA, we have tested the intracellular ROS level produced by H_2_O_2_ and PA–H_2_O_2_ combination. Results exhibited that 200 μM H_2_O_2_ produced 2.5-fold ROS with respect to control. Interestingly PA cotreatment completely blocked the H_2_O_2_-induced ROS. The ROS level produced by PA regardless of concentrations was the same as that of the control. This is also supported by the axonal degeneration apparent from H_2_O_2_ treatment (Figure S3). The microscopic observation of DRG cells using MitoSOX dye determines the superoxide radical, ROS, localized to mitochondria.^38^ We investigated the mitochondria superoxide radical in CDDP with/without PA-treated DRG cells. Results exhibited that CDDP produced a 2.6-fold higher MitoSOX signal than control. It was reduced significantly with PA cotreatment (*P < 0.05; Figure S4).

Mitochondria are the powerhouse of the cell; their maintenance and homeostasis are changed in neurons when subjected to anticancer drugs. CDDP is considered a compound that can destabilize the mitochondria by changing MMP and increasing ROS.^39^ MMP measurement was performed by labeling mitochondria with MitoTracker Orange CMTMRos to study if PA could prevent CDDP-induced mitochondrial damage in DRG. Results determined that CDDP reduced the signal of CMTMRos in cells (Figure 8). Interestingly the CDDP–PA combination was found to enhance the orange CMTMRos signal significantly. This suggests an enhancement of mitochondrial content in the DRG cells, overall attributed to the PA’s role of mitochondria protection.

We are further curious to know whether PA can protect the axons against other platinum derivatives, such as oxaliplatin. Oxaliplatin is a potent anticancer drug that has been used for the treatment of a wide range of cancers.^40^ However, it has been shown to induce peripheral neuropathy in the acute and chronic sensory forms. Oxaliplatin alone and in combination with PA were administered to 5-day-old DRG cells. Any changes in the phenotype were evaluated microscopically. Results found that the cell bodies treated with oxaliplatin alone were fragmented and squeezed (Figure S5 left, arrow), and axons observing were disintegrated and retracted (Figure S5 left, arrowhead). However, combinatory treatment found that the cell bodies remained intact with typical triangular-/oval-shaped soma and a well-integrated network of axons (Figure S5 right). These results indicate that PA may protect the sensory neurons against oxaliplatin-induced degeneration as well.

## 3 Discussion

Platinum-based drugs are ideal candidates for the treatment of ovarian and testicular cancer patients. The PN often associated with these drugs worsens with increasing cumulative doses in the body, and symptoms persist even after the dose completion.^3^ There is little known about the underlying mechanism behind PNs, but it is widely established that the PN is attributed to the degeneration of peripheral sensory axons. Based on the results of this study, we found that CDDP induced axonal degeneration at doses exceeding 2 μM. CDDP-mediated cellular apoptosis is well documented in the neuron cells.^41^ A study on mice treated with CDDP experienced predominantly axonal degeneration characterized by sustained reduction of the caudal sensory nerve action potential amplitude and reduction of sensory nerve conduction velocity.^42^ Current results show that PA cotreatment retains the axonal length and increases the survivability of CDDP-treated cells significantly (*P* < 0.001). PA has been reported to have many beneficial effects in neuroprotection in Alzheimer’s disease,^43^ homeostasis,^44^ anticancer properties^27^ and inflammation control.^45^ Absorption of excessive iron, a significant factor in causing Alzheimer’s disease, was the reason behind neuroprotection.^43^ However, there are no specific reports about PA having a protective effect against the CIPN as we know so far. PA concentrations of up to 1.8 mM have shown compatibility with DRG neurons while treating 24 h after seeding (Figure 1). However, increasing the concentration from 1.8 mM, axon lengths were noticed to be significantly reduced (*P* < 0.001). The platinum-based drugs are characterized by acute and chronic sensory effects via accumulation of the drug into the DRG cells.^46^ We cultured DRG cells for 5–7 days until the axonal network fully covered the wells prior to the neuronal survivability study in response to CDDP or/and PA. CDDP and/or PA were administered to 5-day-old neurons and continued for 45 days to resemble the axon network of CIPN that often occurs in adults. The enhanced protection of the neuronal cell body and axonal network by coadministration of CDDP and PA highlights PA’s role in neuroprotection. CDDP and oxaliplatin share the same cancer cell death pathway by forming covalent DNA adducts and DNA damage.^39^ However, they differ in their neurotoxic effects. Oxaliplatin causes acute and chronic PN in contrast to CDDP, which only causes chronic PN.^46^ Preliminary results suggest that PA preserves the neurons against oxaliplatin-induced degeneration (Figure S5). The protection of axons and cell bodies based on valid neuron networks may confirm PA’s extraordinary neuroprotection role in a wide potential window against CDDP. In the meantime, PA showed synergistic activities with CDDP to kill cancer cells. Pain-relieving agents such as duloxetine often used in PN do not interfere with the anticancer effect of anticancer drugs.^47^ It is noteworthy that PA itself exhibited a reduction of SKOV-3 cells’ viability at doses exceeding 0.9 mM. This is a consistent finding with other reports.^27,48^ Despite the effectiveness of CDDP in killing cancer cells, it is common that the effectiveness decreases over time, which ultimately stops killing the cells due to cancer cell adaptation and evolved resistivity.^49^ This phenomenon makes the eradication of cancer in patients a big challenge. The combined therapy of varying anticancer drugs has been considered a better approach to treat cancers by multiple targets to avoid chances of anticancer drug failure or future cancer relapses.^50^ The treatment with CDDP and PA could be helpful in achieving an effective output. The significant difference in the cell viability between the CDDP/PA cotreatment and its corresponding individual testing strongly suggests that CDDP at low concentrations could be enough if treated in a combinatory manner. Chemotherapeutics are equally toxic to normal cells and function as they are to cancer cells. However, the use of a low concentration of anticancer drugs is highly encouraging in cancer therapy to avoid nonspecific toxicity.

**Figure 7:**
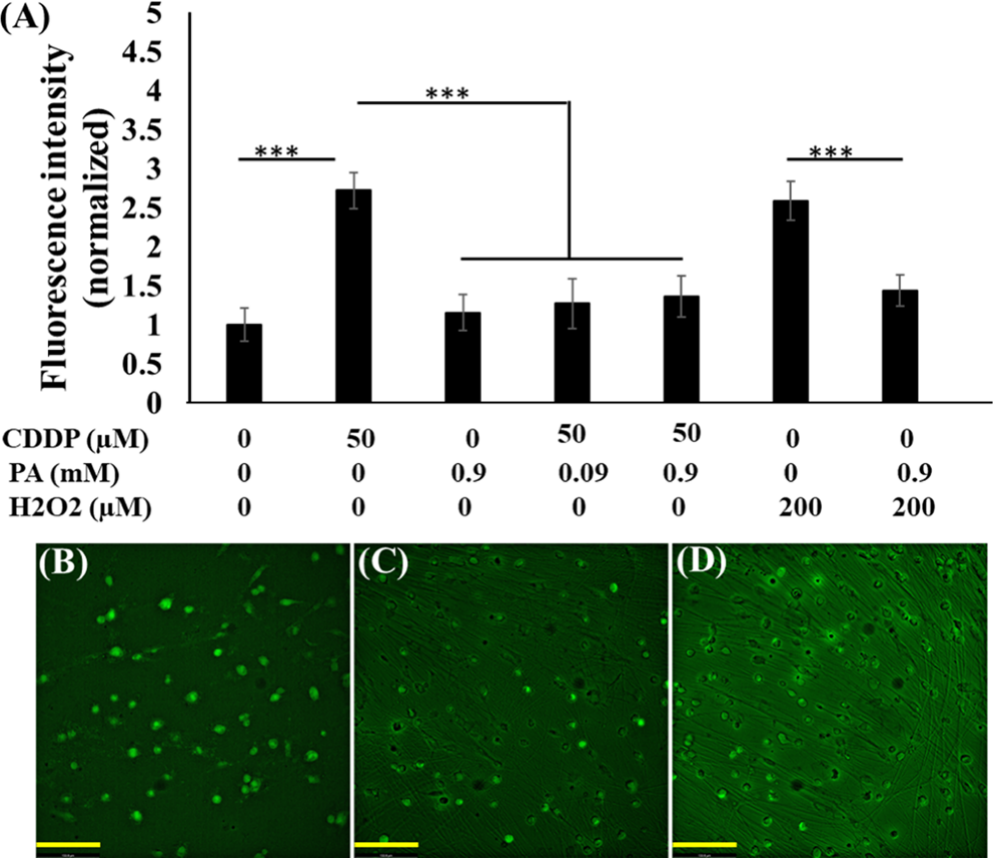
Intracellular ROS assessment on DRG cells. The graph shows the fluorescence intensity measurement of the cells stained and treated with different compounds after exposure to DCFHDA (A). Fluorescence images were acquired by fluorescence microscopy (20×). ImageJ software was used to measure the cellular fluorescence intensity. The data are shown as the means ± SE of three independent experiments. ***P < 0.001. Representative fluorescence images of the DRG cells stained with DCFHDA following CDDP50 (B), PA0.9 (C), and CDDP50PA0.9 (D) treatment. The scale bar is 133.8 μm.

**Figure 8:**
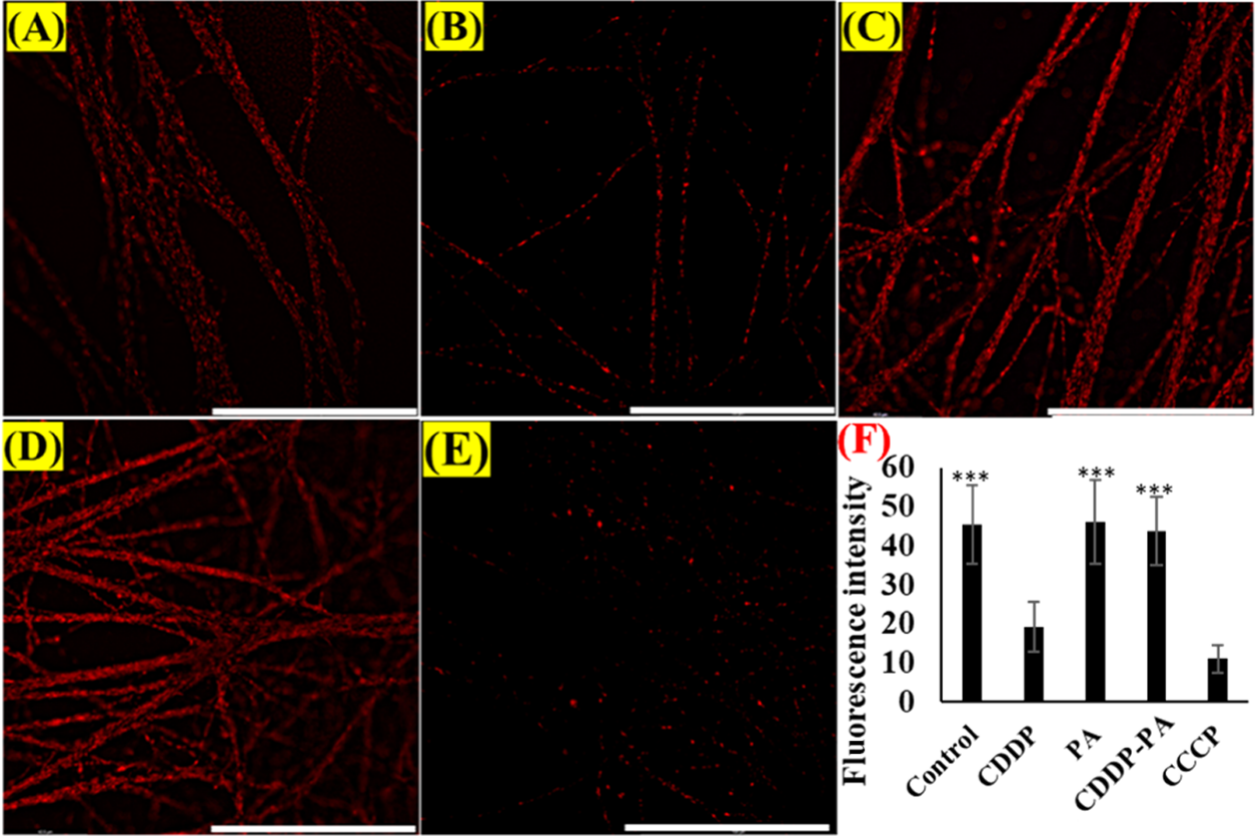
MMP assessment on DRG cells. The drug-treated cells were stained with MitoTracker Orange and examined by fluorescence microscopy (63×) and subsequent intensity measurement by ImageJ. (A)–(E) represent the mitochondria signals belonging to the control, CDDP50, PA0.9, CDDP50PA0.9, and CCCP (50 μM)-treated cells, respectively. The graph shows the fluorescence intensity of MMP (F). Cells were cultured for 5 days, followed by treatment of drugs for 24 h. The MitoTracker Orange dye was used to stain the mitochondria. The fluorescence signal of live mitochondria is proportional to the MMP. The results are expressed as mean ± SE, *n* = 3. ***P < 0.001 compared to CDDP. The scale bar is 100 μm. As a negative control, CCCP was used to treat cells.

**Figure 9:**
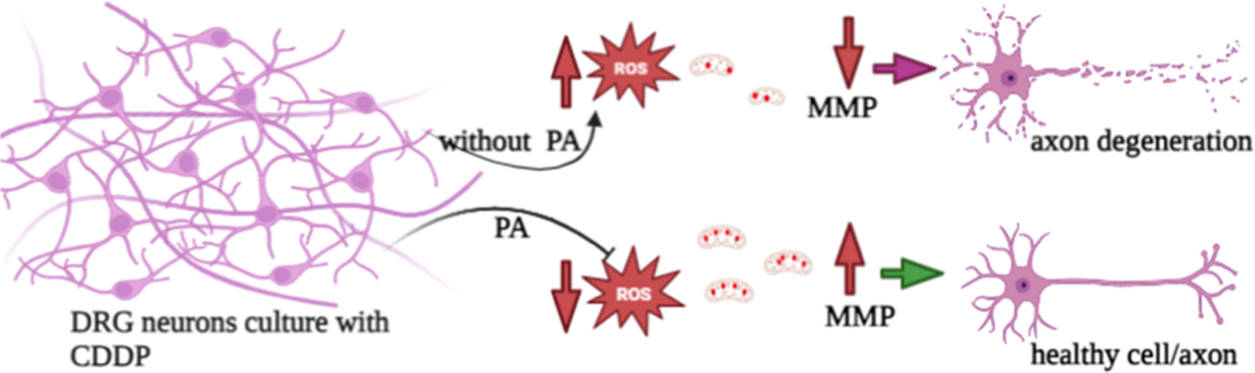
Proposed PA-mediated neuroprotection mechanism. The illustration shows the proposed mechanism of neuroprotection against CDDP-induced axon degeneration. Biorender software was used partially to draw outlined figures.

The current study showed that CDDP increased intracellular ROS generation, while MMP was found to be reduced in DRG neurons. This is consistent with increasing recognition of excessive production of ROS, depleting mitochondria population, and disruption of mitochondrial bioenergetics upon oxaliplatin administration.^20^ The treatment with PA completely inhibited the ROS signals and significantly retained the MMP (Figures 7 and 8). As a positive control, 200 μM H_2_O_2_ was introduced into the cell culture, which contributed to a higher ROS level, but PA cotreatment scavenged the ROS completely, strongly supporting the claim that PA serves as an ROS scavenger. H_2_O_2_ is a less reactive ROS, which does not produce green fluorescence signals when reacting with DCFH molecules.^51^ Nevertheless, it forms an extremely reactive hydroxyl radical when it is in contact with other metal ions and produces strong green signals when it is in contact with DCFH. The corresponding microscopy images showed that cells were aggregated following the administration of 200 μM H_2_O_2_ (Figure S3). However, H_2_O_2_–PA-treated cells retained the cell phenotype, cells were observed to be healthy, and the axonal network remained intact. Accumulation of ROS-induced oxidative stress causes mitochondrial damage.^52^ It was also found that mitochondrial superoxide increased in DRG cells upon the treatment of CDDP (Figure S4). Superoxide radicals are highly reactive and cause damage to the mitochondrial inner structure.^53^ Interestingly, coadministrated CDDP–PA reduced the superoxide signals significantly. PA itself had a remarkably lower superoxide signal than CDDP or CDDP–PA. PA has a high binding ability to radicals by donating electrons or hydrogen atoms, thereby neutralizing the reactivity.^35^ Hence, ROS formation may have been blocked by PA through scavenging of the radicals that could help maintain mitochondrial health. On the other hand, mitochondria are the largest contributor to the cellular ROS.^54^ PA protected the mitochondria, thereby simultaneously inhibiting the mitochondria-mediated production of excessive superoxide and overall intracellular ROS. Growing evidence suggests that CDDP increased ROS resulting in DNA damage and neuronal cell death.^37^ Mitochondria play a key role in neuroprotection from injuries by maintaining biogenesis.^55^ Reduced levels of superoxide radicals and increased levels of the MMP of PA-modulated cells compared to CDDP-treated ones affirm the relief of the cells from oxidative stress and confirm mitochondrial protection by PA (Figure 9).

Inositol, a derivative of PA, is present in the blood in its free form, typically about 29 μM concentration, but likely the concentration changes in response to dietary conditions. Inositol is one of the most abundant metabolites in the brain with concentrations up to 5.1 mM, a 200-fold higher concentration than in the plasma.^56^ In one study, PA up to 4 wt % in water was treated daily for up to 15 weeks without limiting intake in the prostate cancer model mouse.^57^ They found that IP6 inhibited tumor angiogenesis and tumor growth remarkably. These facts show that a large influx of PA could be needed to achieve enhanced therapeutic effects, including anticancer and neuroprotection effects. We found that the increased SKOV-3 cell death with PA concentrations above 0.9 mM, while there was no effect on SKOV-3 cells at lower concentrations. The selective accumulation of the compounds at such concentrations into cancerous tissues by systemic administration is impossible. However, the local delivery of PA into the targeting cancerous tissue could be an option to achieve enhanced cancer cell death. The most common sources of PA are legumes, whole grains, and milk.^25,26,58^ The world population, including cancer patients, consumes PA on a daily basis from secondary sources. However, the positive effect of PA in a clinical setting has not been well documented as a neuroprotective compound. The thorough study of optimal dose calculation, scheduling, and confirming the toxicity profile of CDDPs with PA is necessary to carry out in animal models first. Similarly, the molecular mechanism associated with PA-induced cancer cell death and simultaneous neuroprotection when combined with CDDP will be necessary to study. Moreover, the consequences of the PA treatment with other anticancer drugs including paclitaxel, vincristine, and bortezomib, which mostly account for CIPN, will also need to be studied to determine further increasing acceptance to treat PN. CIPN can occur by a variety of mechanisms, not only DRG damage. Herein, an in vitro study of DRG cells by PA against CDDP-induced PN may not be sufficient to determine whether PA is effective in an in vivo CIPN model. Similarly, the evaluation of neuroprotection in embryonic cells certainly limits the scope of PA in the context of CIPN, which often occurs in adult neurons. A study focusing on overcoming these limitations could be a part of a future study. The overall findings of this study strongly suggest that PA could not only serve as a better option to manage CIPNs but also may have the ability to protect against neurotoxicity caused by heavy metal ions and other neurodegenerative conditions. However, further research is necessary to support such assumptions.

## 4 Materials and Methods

### 4.1 Materials

CDDP and oxaliplatin were purchased from Merck, NC. PA (50% purity) was purchased from Acros Organics, NC. Fluorescent MitoTracker Orange CMTMRos was obtained from Thermo Fisher Scientific. Similarly, poly-d-lysine (PDL) and laminin were purchased from Sigma-Aldrich. 100× penicillin/streptomycin (P/S), 100× glutamate, B27 supplement, nerve growth factor (NGF), and 5-fluoro-2′-deoxyuridine thymidylate synthase inhibitor (FUDR) were received from Sigma-Aldrich, NC. ROS detection reagent 2′,7′-dichlorofluorescein diacetate (DCFHDA) was purchased from Sigma, NC. 3.3 mM CDDP stock (3.3 mM) was prepared in normal saline (0.9% NaCl (w/w)).

### 4.2 Dorsal Root Ganglia (DRG) Neuron Extraction and Culture

All experiments related to animals were conducted in accordance with protocols approved by the Institutional Animal Care and Use Committee (IACUC) of the University of North Carolina Charlotte. The embryos were harvested from E15 pregnant Sprague-Dawley rats under the flow of 7.5 L/min isoflurane. The embryos were later dissected using a stereoscopic dissection microscope (Nikon SMZ745T, Japan). The DRG cells were collected by dissecting the spinal column and later dissociated, as reported earlier in our report.^59,60^ Briefly, 2 mL of DRG clumps containing L15-P/S media were incubated with 1 mL of collagenase (10 mg/mL in L15-P/S) and 1 mL of DNase (0.5 mg/mL) for 30 min, followed by centrifugation. The cells were further treated with 0.25% trypsin for 5 min at 37 °C, quenched with fresh medium containing fetal bovine serum (FBS), and centrifuged to collect the DRG cells. The cells were then resuspended in a DRG culture medium at a density of 500,000 cells/mL and seeded on the 96-well plate (200 μL) unless otherwise stated. The DRG culture medium consisted of a neurobasal medium supplemented with 1% 100× P/S, 1% glucose, 100× 2% glutamate, 2% B27 supplement, and 20 ng/mL NGF. FUDR (13 μg/mL) was added to culture media for the elimination of possible non-neuronal glial cells in culture. The cell culture was maintained by replenishing the half-well media every 72 h.

### 4.3 Axon Length Measurement and Neuronal Survival

The effect of PA on neurons alone or against CDDP-treated DRG cells was studied in terms of axon length changes. 200 μL of medium containing 1000 DRG cells was seeded on each PDL/laminin-coated 96-well glass-bottom plate. After 24 h of culture, the cells were treated with CDDP, PA, and a combination for another 24 h. PA was administered across a range of concentrations, including 0.002, 0.02, 0.09, 0.9, 1.8, 4.5, and 9 mM. CDDP was used at concentrations of 1, 2, 5, 10, 20, and 50 μM in a separate set of experiments. After 24 h of drug treatment, the cells were stained with 5 μM calcein-AM dye (Corning)-containing media, followed by live-cell imaging by a fluorescence microscope (Leica DMi8, Germany). ImageJ software (Java 1.8.0_345 (64-bit)) was used to estimate the length of the axons. At least 60 axons were selected from triplicate samples to calculate the average axon length. To evaluate neuronal survival, the cells were cultured for 7 days to get axon confluence in wells. Later, the DRG cells were subjected to culture under CDDP, PA, and CDDP–PA for 7 days. The cell survivability was evaluated by counting the live cells and determining the percentage of cells relative to the control group, which was considered 100%. Three frames of each well of triplicate samples were used to estimate the average numbers.

### 4.4 Effect of PA on CDDP’s Anticancer Properties

The human ovarian cancer cell line SKOV-3 was used as an in vitro model in this study to identify the effect of PA on the anticancer ability of CDDP. The SKOV-3 cells were seeded onto the 96-well plate at a density of 5000 cells/well. After 24 h of cell seeding, CDDP or/and PA of different concentrations were added into the wells-containing cells. Following 48 h, the cells’ nucleus was stained with 10 μM Hoechst staining (Sigma) according to the manufacturer’s protocol. Later, the cells were imaged using fluorescence microscopy, and cellular nucleus counting was conducted by ImageJ. To count the nucleus, the following steps were carried out; Open image, Click Image type to 16 bits, Click Image and Adjust> Click Image and Threshold, Click Process > Click Make Binary > Click Process > Click Convert to mask > Click Process > Click Watershed, Click Analyze particles. and Click Ok. Set size pixel∧2 to 10-infinity at the analyze step. This step ignores the small dots and does not count them as particles (nucleus). The cell viability was calculated as a percentage considering the average count for the control as 100%. Each group of treatment comprised triplicate wells.

### 4.5 Measurement of Intracellular ROS Production and Superoxide Radical

Intracellular ROS generation in response to drug exposure was measured using the cell-permeable ROS detection reagent DCFDH. DCFDH is a nonfluorescent probe that can freely diffuse into the cells where it converts it to 2′,7′-dichlorofluorescein (DCF) by several ROSs.^51^ 5-day DRG cells on 96-well plates were treated for 24 h with different combinations of PA/CDDP. Cells were washed with warm phosphate buffer saline (PBS, pH 7.4) and incubated with 20 μM DCFDH at 37 °C for 15–30 min. The cells were then washed once with PBS, followed by a medium without phenol red. The intracellular fluorescence signals were captured by a fluorescence microscope. The fluorescence signals of the equal area (100 μm × 100 μm) for different samples were estimated by ImageJ software. As a positive control, cells treated with 200 μM hydrogen peroxide (H_2_O_2_) alone were used. PA’s combination with H_2_O_2_ may ensure the role of PA in inhibition or excitatory effect on ROS. To measure the mitochondria-specific superoxide radicals, MitoSOX green (Thermo Fisher, catalog M36006) dye was added to drug-treated cells according to manufacturer instructions. Briefly, the cells were washed twice with warm Hank’s balanced salt solution (HBSS 1×, Gibco). Later, 50 μM MitoSOX green in HBSS solution along with 10 μM Hoechst staining as a counter stain was added to the cells and incubated for 30 min before imaging by fluorescence microscopy at 63× magnification. The mean fluorescence signal in the cell body was measured for cells treated with compounds. Ten cells from each triplicate sample were used to estimate the mean fluorescence signal.

### 4.6 Analysis of Mitochondrial Membrane Potential (MMP)

DRG cells were plated at 1000 cells/well in a glass-bottom 96-well plate and allowed to culture for 5 days. Later, the cells were treated with CDDP, PA, or both for 24 h. The cocultures were stained with MitoTracker Orange CMTMRos (100 nM, Thermos Fisher, NC) for 30 min at 37 °C according to the manufacturer protocol. As a negative control, neurons were treated with 50 μM carbonilcyanide *p*-trifluoromethoxyphenylhydrazone (CCCP, Sigma-Aldrich), a mitochondrial uncoupler, for 15 min followed by staining. CCCP is a well-known mitochondrial destabilizing agent that increases proton permeability and MMP.^61^ The cells were then imaged using inverted fluorescence microscopy on a DMi8 Leica Confocal Microscope (Leica Microsystems, Germany) with a 63× objective, and images were analyzed with ImageJ software. The MitoTracker Orange CMTMRos is an orange fluorescence dye that stains the mitochondria, specifically in live cells, and its accumulation is proportional to the MMP.^62^

### 4.7 Statistical Analysis

All data were presented as the mean ± standard error (SE, *n* = 3). The probability value (*P*-value) between the groups was analyzed by using one-way analysis of variance (ANOVA) followed by post doc Tukey’s test for multiple comparison. A *P*-value of less than 0.05 is considered statistically significant.

## 5 Conclusions

We have shown for the first time that PA effectively protects DRG neurons and promotes neuronal stability by mitigating the degenerative impact of cisplatin and oxaliplatin. The administration of PA did not compromise the effectiveness of CDDP in its anticancer activity; instead, it exhibited a synergistic effect when applied in combination. It was found that PA combination with CDDP reduced the mitochondria superoxide radicals by 1.5-fold and intracellular ROS by 2.7-fold. These ROS scavengings could be considered in relation to enhanced neuroprotection by PA against CDDP-induced peripheral neuron degeneration. Enhanced MMP by 2.6-fold in CDDP–PA-treated cells compared to that in CDDP alone further affirms the mitochondrial protection by PA. CDDP is often known for DNA damage and cell death. However, this study did not delineate the mechanism of synergistic cancer cell death, and hence, this study warrants further investigation.

## Supporting Information Available

Phase contrast and fluorescence images of CDDP treatment for 2 weeks; phase contrast images of SKOV-3 treated with different drug combinations; phase contrast images of DRG neurons treated with H_2_O_2_ and PA-H_2_O_2_; mitochondrial superoxide measurement; and phase contrast images of the DRGs treated with oxaliplatin and PA cotreatment (PDF)
