# Supplementary material for: Phytic Acid Maintains Peripheral Neuron Integrity and Enhances Survivability against Platinum-Induced Degeneration via Reducing Reactive Oxygen Species and Enhancing Mitochondrial Membrane Potential
Source: ACS Chem Neurosci. 2024 Mar 6;15(6):1157–68. doi: 10.1021/acschemneuro.3c00739 (PMC10958516; doi:10.1021/acschemneuro.3c00739)
Supplement: Supplementary file 1 — cn3c00739_si_001.pdf [file cn3c00739_si_001.pdf]

# Supporting information for

## Phytic acid maintains peripheral neurons integrity and enhances survivability against platinum induced degeneration via reducing reactive oxygen species and enhancing mitochondrial membrane potential.

Arjun Prasad Tiwari[1], Bayne Albin[1], Khayzaran Qubbaj[1], Prashant Adhikari[1], In Hong Yang[1]*

[1]Center for Biomedical Engineering and Science, Department of Mechanical Engineering and Engineering Science, University of North Carolina at Charlotte, Charlotte, North Carolina 28223, United States

* Corresponding authors: In Hong Yang; E-mail: iyang3@charlotte.edu

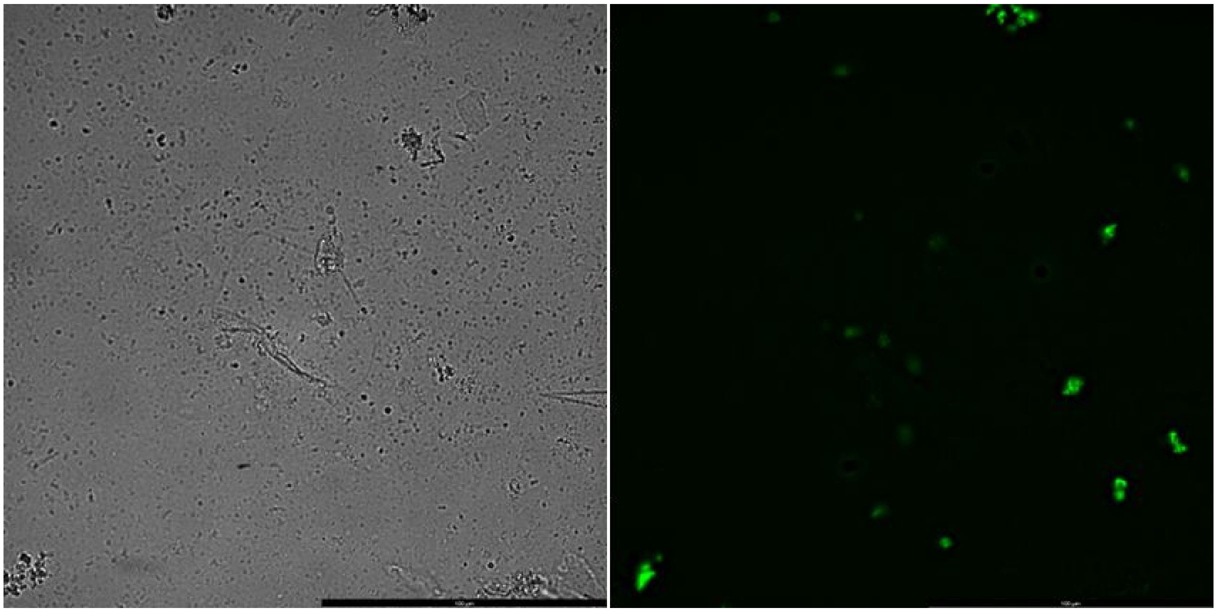

***Figure S1.*** *Phase contrast and fluorescence images of CDDP10μM treatment for 2 weeks.*

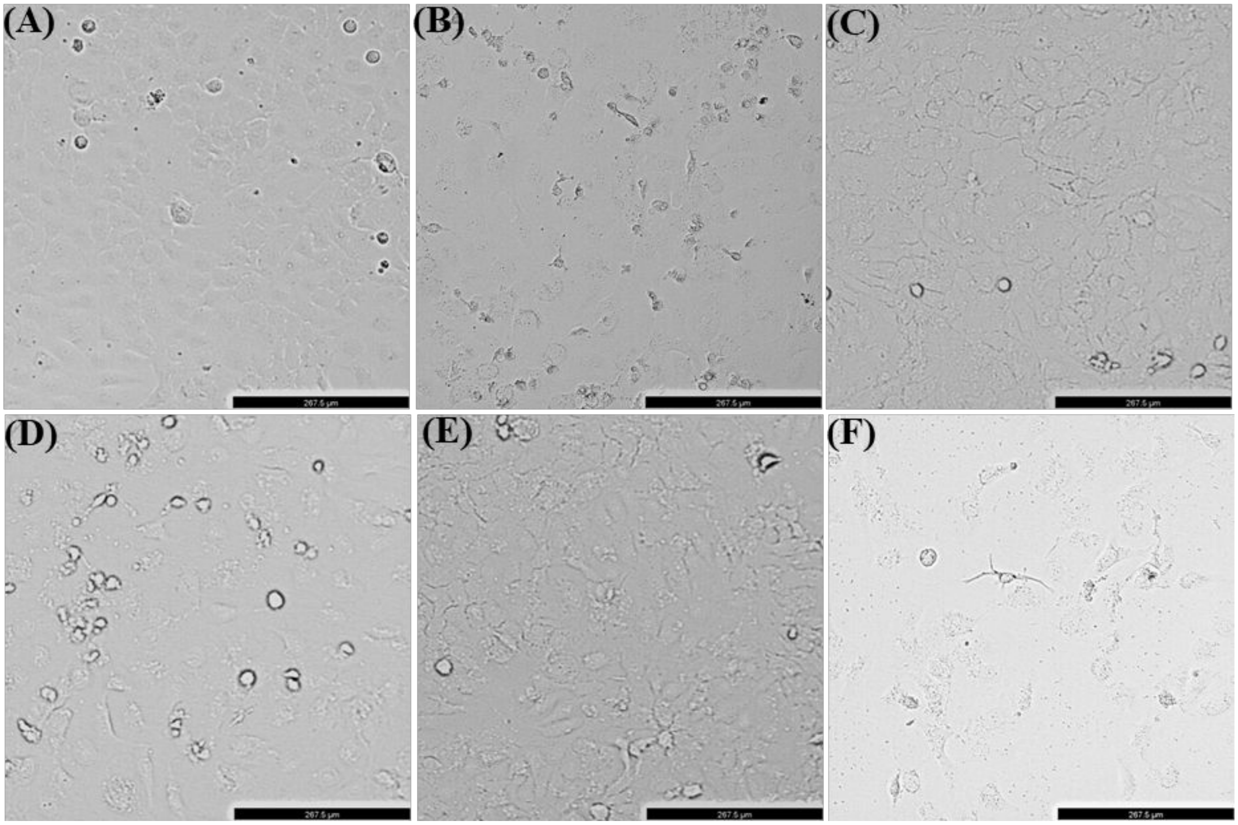


***Figure S2.*** *Phase contrast images of SKOV-3 treated with different drug combinations Control (A), CDDP10 (B), PA0.9 (C), CDDP10PA0.9 (D), PA1.8 (E), and CDDP10PA1.8 (F). The number along with CDDP represents µM concentration and the number along with PA represent mM range. Bar scale is 267.5 µm.*

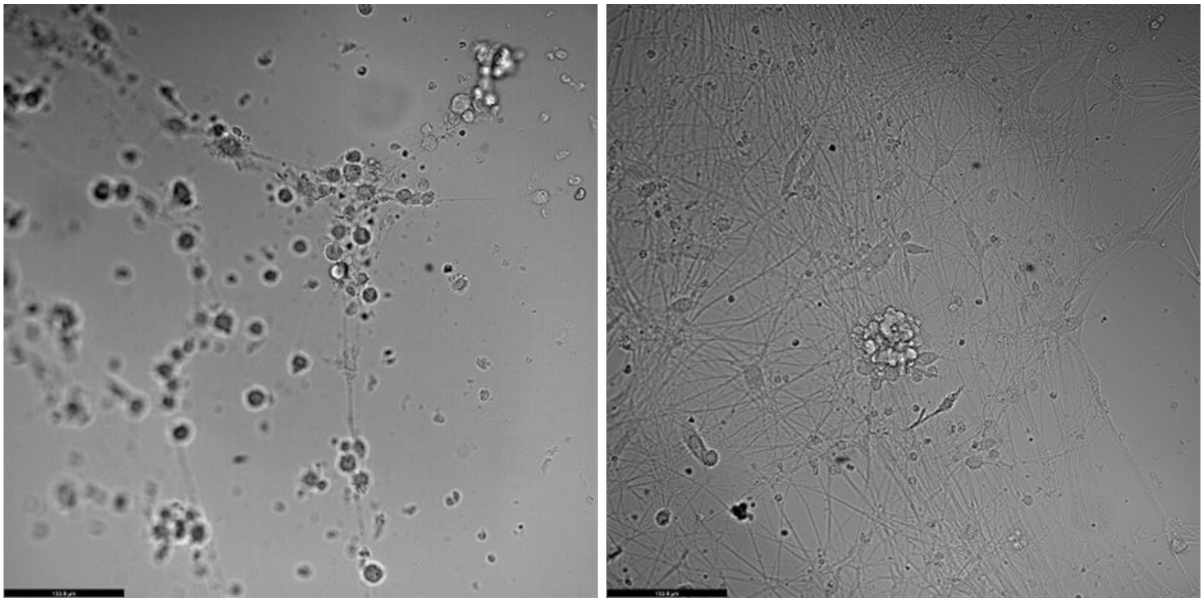

***Figure S3.*** *Phase contrast images of DRG neurons treated with* $H_2O_2$ *(200μM) (left) and PA-*$H_2O_2$ *of 0.9 mM and 200 μM, respectively (right), 24 h. 5-day old DRGs were treated with designated compounds for 24 h.*

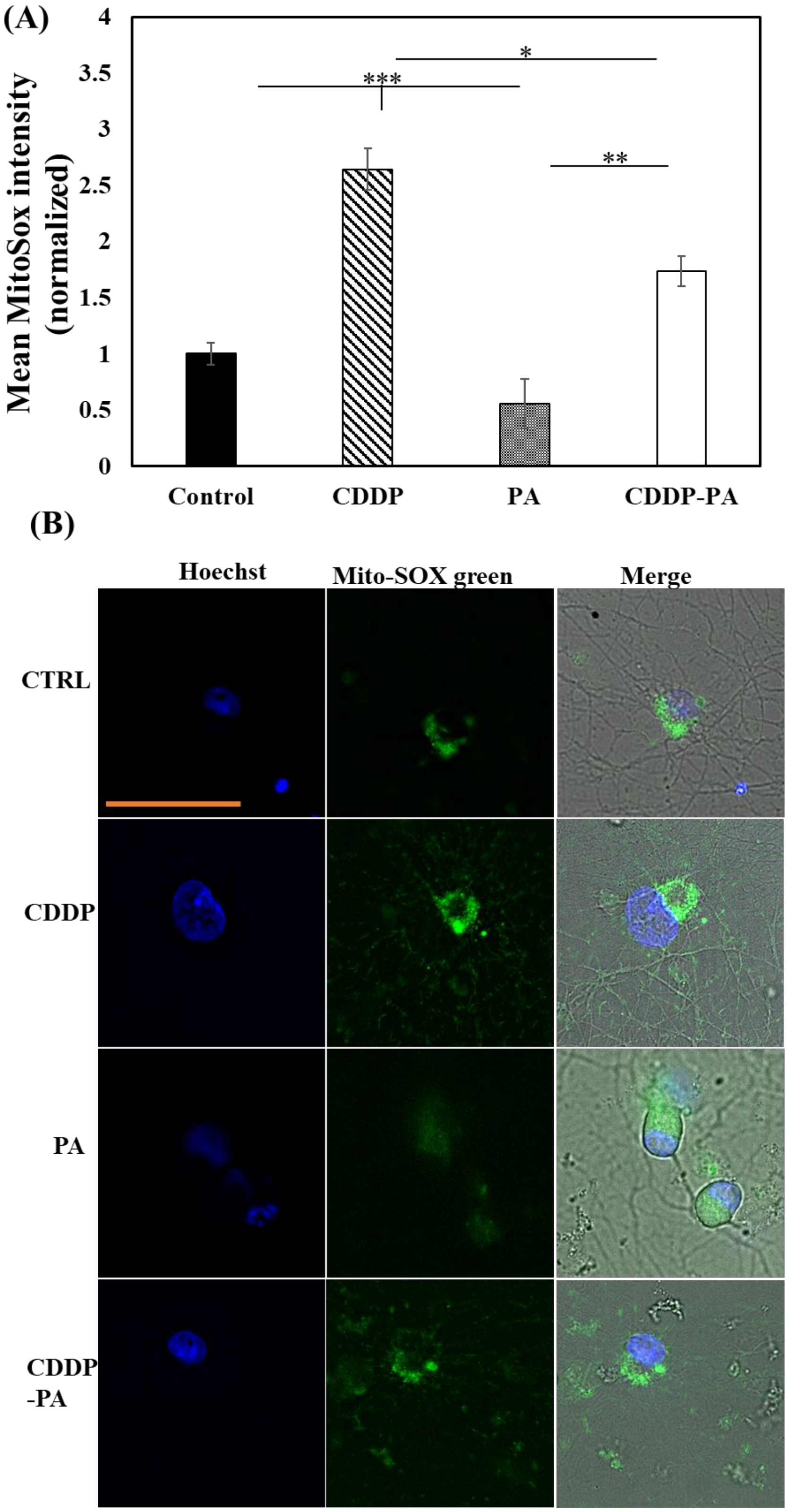

(A)
Mean MitoSox intensity (normalized)
4
3.5
3
2.5
2
1.5
1
0.5
0
*
***
**
Control
CDDP
PA
CDDP-PA
(B)
Hoechst
Mito-SOX green
Merge
CTRL
CDDP
PA
CDDP -PA

***Figure S4.*** *Mitochondrial superoxide measurement: (A) mean fluorescence intensity of MitoSOX after the treatment of CDDP50, PA0.9 and combination. (B) florescence image of the MitoSOX signal of DRGs treated with vehicle (control), CDDP50, PA0.9 and combination. *, ** and *** represent the P value <0.05, <0.01 and <0.001, respectively. The cells were treated for 4 h.*

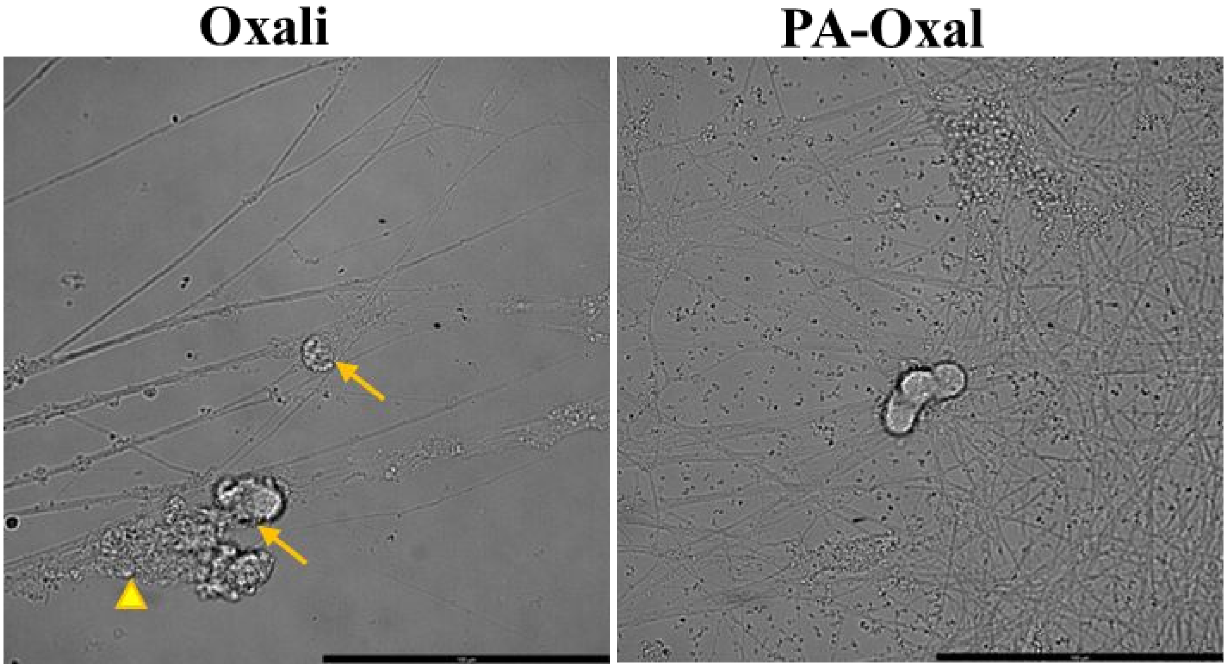


***Figure S5.*** *Neuroprotection against Oxaliplatin induced axons degeneration. Phase contrast images of the DRGs treated with oxaliplatin (10µM) and PA-cotreatment (Oxaliplatin 10µM PA 0.9 mM) on 5-days old DRGs cells. The drugs were treated for another 72-h followed by image taken (63X) Scale bar is 100 µm.*
